# Development of an Immunochromatographic Strip for Rapid Detection of Mink Enteritis Virus

**DOI:** 10.3389/fmicb.2022.839320

**Published:** 2022-03-09

**Authors:** Peng Lin, Jianke Wang, Shanshan Song, Yuening Cheng, Li Yi, Shipeng Cheng, Zhenjun Wang

**Affiliations:** ^1^Institute of Special Animal and Plant Sciences, Chinese Academy of Agricultural Sciences, Changchun, China; ^2^Hebei Veterinary Biotechnology Innovation Center, College of Veterinary Medicine, Hebei Agricultural University, Baoding, China; ^3^College of Veterinary Medicine, Jilin University, Changchun, China

**Keywords:** immunochromatographic strip (ICS) assay, diagnosis, mink enteritis virus, parvovirus, canine parvo virus

## Abstract

Although mink enteritis virus (MEV) is an acute, virulent, and highly contagious pathogen in minks, there is currently a lack of a quick diagnostic method. By conjugating colloidal gold nanoparticles with the MEV-specific monoclonal antibody, monoclonal antibody (MAb) 14, we developed a single-step competitive immunochromatographic strip (ICS) assay for simple determination of MEV. The optimal concentrations of the colloidal gold-coupled MAb 14 (coating antibody), the capture protein (MEV VP2 protein), and the goat anti-mouse antibody were 1.0, 0.8, and 1.0 mg/ml, respectively. The limit of detection was approximately 512 hemagglutination units/100 μl of MEV B strain. Other common viruses of mink were tested to evaluate the specificity of the ICS, and the results showed no cross-reactivity for other pathogens. In comparison with the Anigen Rapid canine parvovirus (CPV) Ag Test Kit (BioNote, Korea) in testing 289 samples, the percentage of agreement and relative sensitivity and specificity of the MEV ICS assay were 94.1, 93.2, and 97.1%, respectively. The ICS test was found to be a sufficiently sensitive and specific detection method for the convenient and rapid detection of MEV.

## Introduction

Mink enteritis virus (MEV) can cause acute, virulent, and highly contagious disease in minks and is characterized by acute hemorrhagic enteritis and leucopenia, especially in younger animals. The virus belongs to the genus *Protoparvovirus* (subfamily, *Parvovirinae*; family, *Parvoviridae*). This genus also includes canine parvovirus (CPV), feline panleukopenia virus, and raccoon parvovirus. The capsid proteins of these four viruses share 90% similarity (Parrish and Carmichael, 1983). With an average diameter of 18–24 nm, MEV is a nonenveloped ssDNA virus consisting of two large open reading frames (ORFs), which encode two nonstructural proteins (NS1 and NS2) and two structural proteins (VP1 and VP2) through alternative splicing of the mRNAs. VP2 is the main viral capsid, and it comprises approximately 90% of the capsid. Mink viral enteritis caused by MEV was first reported by Schofield in 1947 in Canada (Schofield, 1949) although the isolation and identification of the viral pathogen did not occur until 1952 (Wills, 1952). And now the disease affects mink farming worldwide and has become one of the most significant threats to mink production in some areas (Steinel et al., 2001).

Currently, PCR, ELISA, hemagglutination and hemagglutination inhibition (HA-HI), loop-mediated isothermal amplification, etc. are used for MEV detections. Although these methods have a high sensitivity and specificity, they are time-consuming, expensive, and mostly restricted to well-equipped laboratories (Rivera and Sundquist, 1984; Zhang et al., 2007; Wang et al., 2011, 2012, 2013). In recent years, the colloidal gold-based immunochromatographic strip (ICS) assay has been expanded to several applications in various fields of scientific research, particularly in the clinical diagnosis and detection of viruses, bacteria, pesticides, and medicine residues (Zhu et al., 2008; Li et al., 2010; Preechakasedkit et al., 2012; Wang et al., 2019). The ICS is a rapid, safe, and convenient method based on specific antigen–antibody reactions (Jiang et al., 2011). To date, MEV has not yet been detected using a colloidal gold-based ICS assay.

Here, we describe the development of a single-step competitive ICS-based assay for rapid clinical detection and epidemiological characterization of MEV. And the ICS assay was also used in the detection of CPV infection in dogs.

## Materials and Methods

### Strains and Cells

The MEV B strain (MEVB), canine distemper virus (CDV)-3, CPV-BJ-21, canine adenovirus (CAV)-2c, and *Aleutian mink disease virus* (AMDV) strains were obtained from the Institute of Special Animal and Plant Sciences, Chinese Academy of Agricultural Sciences (Lin et al., 2018). F81 and SP2/0 cells were purchased from the American Type Culture Collection (Manassas, VA, United States). F81 cells were cultured in modified Eagle’s medium containing 6% fetal calf serum (BI, Kibbutz Beit Haemek, Israel), while SP2/0 cells were cultured in RPMI-1640 medium (Hyclone, Logan, UT, United States) containing 10% fetal calf serum. All cells were cultured at 37°C in a CO_2_ incubator.

### Purification of the MEVB Strain

The MEVB strain was purified by ultracentrifugation as previously described (Xu et al., 2014). Briefly, F81 cells infected with MEVB were frozen and thawed, and then centrifuged at 5,000 rpm for 40 min. The supernatants were centrifuged, mixed with 8% PEG-6,000, and then centrifuged at 13,000 rpm for 90 min at 4°C. The pellet was dispersed in 1.0 mM EDTA, added to a sucrose gradient (35–65%), and then centrifuged at 35,000 rpm for 10 h. The virus-containing pellet was collected and resuspended with TB buffer.

### Preparation of Monoclonal Antibodies

A set of monoclonal antibodies (MAbs) specific to MEV was produced from a mouse immunized with the MEVB strain using a previously described protocol (Wang et al., 2009). The fused hybridomas were cultivated in 96-well plates with a feeder layer. Positive hybridomas were determined by indirect ELISA and cloned by limiting dilution in 96-well plates. The MAbs were assayed by indirect ELISA and tested for specificity by indirect immunofluorescence and Western blotting assays.

### Indirect ELISA

Mink enteritis virus-specific antibodies were detected using an indirect ELISA method as previously described (Xu et al., 2014). Briefly, purified VP2 protein (Lin et al., 2015) was coated onto microplates and incubated overnight at 4°C, and the MAbs and goat anti-mouse IgG/HRP (Proteintech, Chicago, IL, United States), which had been diluted to 1:500 and 1:5,000 in 5% skim milk, were incubated at 37°C for 2 h. The absorbance was measured at 450 nm with a spectrophotometer (Biotek Synergy H1, United States).

### Indirect Immunofluorescence Assay

F81 cells were grown in 24-well plates and infected with MEVB at a multiplicity of infection of 0.2. At 36 h postinfection, the cells were fixed with 4% paraformaldehyde for 30 min, blocked in 5% BSA for 1 h, and then probed with MEV MAbs at a dilution of 1:100 for 2 h and anti-mouse FITC-labeled secondary antibody (Sigma-Aldrich, St. Louis, MO, United States) at a 1:200 dilution for 1 h. Finally, the cells were visualized using a fluorescence microscope (Nikon, Inc., Tokyo, Japan).

### Western Blotting

Western blotting was performed as described previously (Lin et al., 2019; Wang et al., 2020) Briefly, purified MEV VP2 protein (Lin et al., 2015) were separated on a 12% SDS-PAGE gel then transferred onto a nitrocellulose membrane (Millipore, Merck KgaA, Darmstadt, Germany), blocked with 5% skim milk and probed with a mouse anti-MEV primary antibody (1:100) followed by a goat anti-mouse HRP secondary antibody (1:5,000, Proteintech, Chicago, IL, United States). Signals were visualized by the Pierce ECL Western blotting substrate (Thermo Scientific, Waltham, MA, United States) for 2 min.

### MAbs Labeled With Colloidal Gold

We prepared 20-nm colloidal gold particles by trisodium citrate revivification, according to a previously described method (Paek et al., 2000). Briefly, chloroauric acid (HAuCl_4_, Sigma, St. Louis, MO, United States) was heated, and 1% trisodium citrate was added until the color stabilized. The solution was mixed with MAbs and buffer solution (0.1% BSA, 2% sucrose, 0.5% PEG-20,000, and 0.2% sodium azide, pH 7.4). The prepared colloidal gold probe was visually observed and authenticated by visual inspection, transmission electron microscopy (TEM), and UV-visible spectrophotometry.

### Assembly of the Colloidal Gold Test Strip

**Figure 1:**
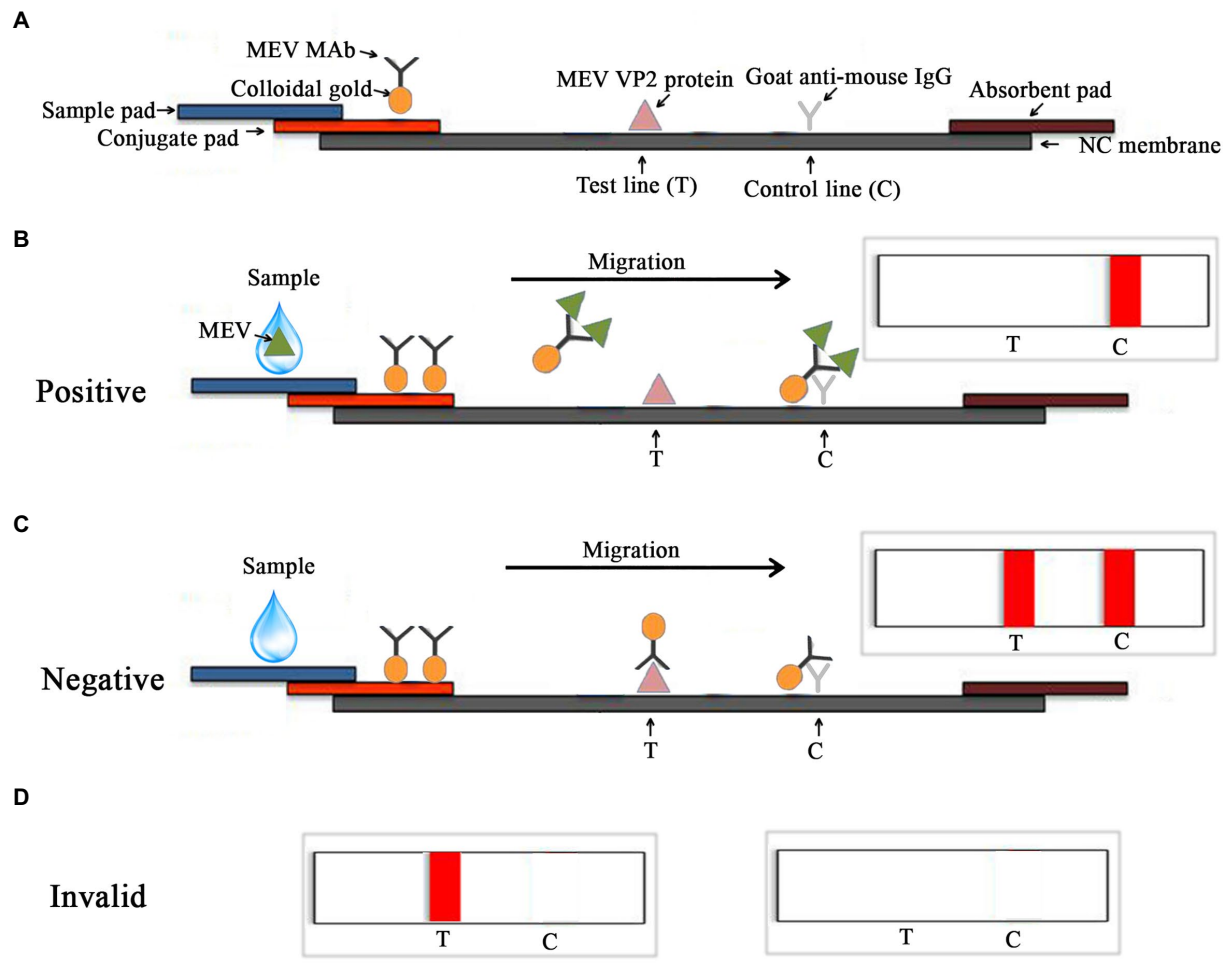
Schematic diagram of the MEV competitive ICS assay. **(A)** Schematic side view of colloidal gold competition and the principle of detection. The sample contained the MEV, the colloidal gold-monoclonal antibody (MAb) 14 conjugates would combine with the MEV. When the antigen–antibody complex migrated to the C line, it would combine with goat anti-mouse IgG, and a band would appear at the C line **(B)**. If two bands were observed at both the T and C lines, the sample was considered negative **(C)**. The test is invalid if only one band appears at T line and no band is present **(D)**.

A nitrocellulose membrane was coated with purified MEV VP2 protein and goat anti-mouse IgG (Bioss, Beijing, China) as the test line (T line) and the control line (C line), respectively. The glass cellulose membrane was used as a sample pad, which was immersed in 0.1 M Tris-buffered saline. The polyester fiber used as the conjugate pad, which was fully immersed in 0.01 M phosphate-buffered saline. Figure 1A shows the principle of the colloidal gold competition test strip for the detection of MEV.

### Performance Test

We used the ICS to test for common viruses of mink, including MEVB, CDV-3, CPV-BJ-21, CAV-2C, and AMDV, with a fecal sample from an MEV-noninfected mink as the negative control to detect specificity. The MEVB strain was used as a positive sample with an *HA titer* up to 16,384 for sensitivity detection. The packed colloidal gold test strips were sealed in a constant temperature humidity chamber for 6 months at 4 and 25°C, respectively. The same samples were tested every month to test their specificity and sensitivity. Therefore, within 2 min, two bands will appear for negative samples (one on the T line and one on the C line), whereas only one band will appear on the C line for positive samples.

### Clinical Application of the ICS

We used the CPV Ag test kit (BioNote, Korea), which can cross-react with MEV, to evaluate the MEV ICS in the study. A total of 289 fecal swabs were collected, 104 specimens were suspected to contain MEV and 185 specimens were suspected to contain CPV, in Heilongjiang, Jilin, Liaoning, Hebei, and Shandong provinces and Beijing in China (signs of infection included anorexia, vomiting, and diarrhea). All the samples were confirmed by PCR (Zhao et al., 2017), 95 were positive for MEV and nine were negative out of 104 mink specimens, 154 were positive for CPV, and 31 were negative out of 185 dog specimens. Each fecal swab sample was added to normal saline, forming a turbid mixture, and it was placed on the prepared colloidal gold test strip for MEV and an antigen rapid CPV Ag test detection, respectively.

## Results

### Characterization of Anti-MEV MAbs

**Figure 2:**
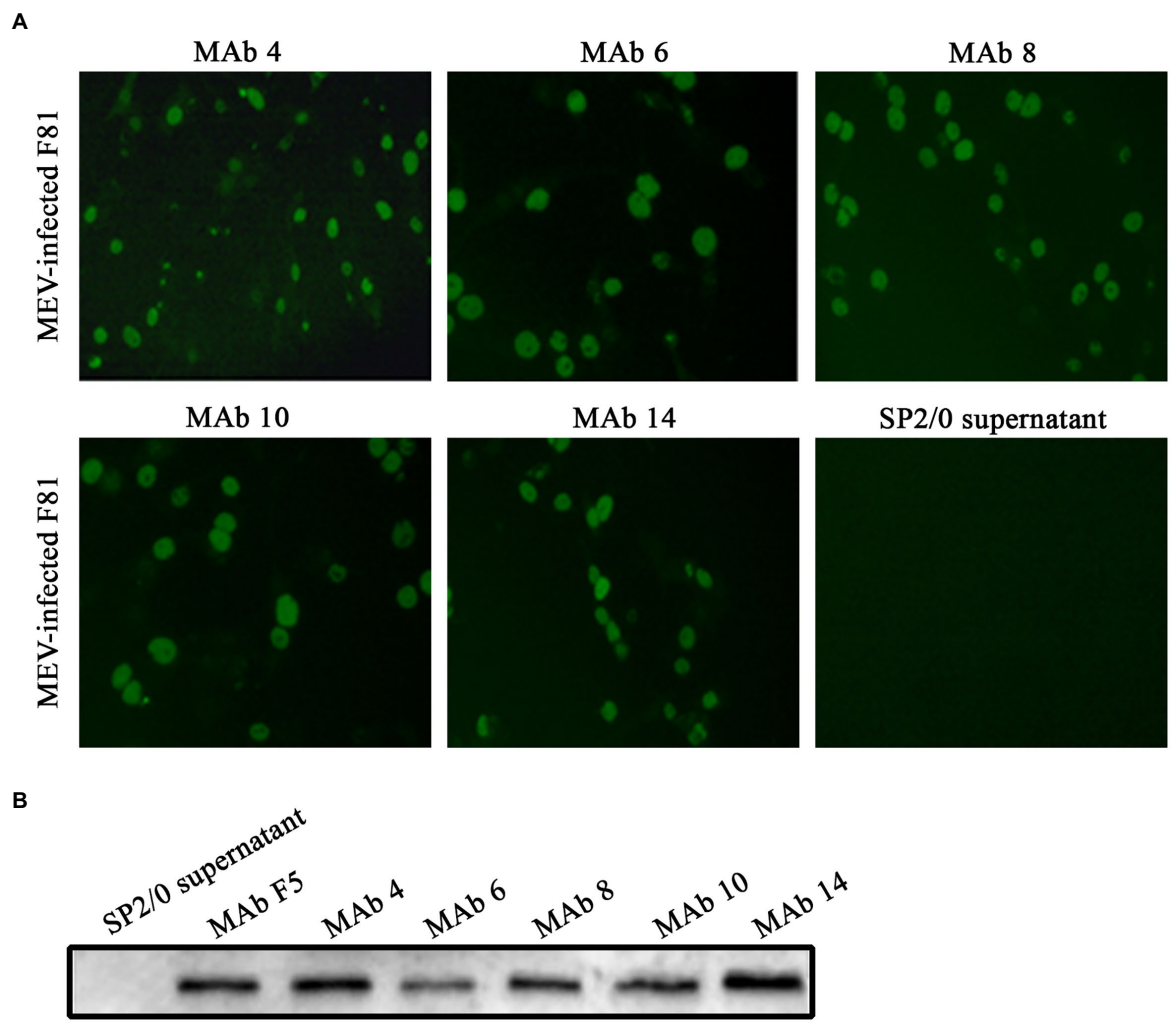
Identification of MEV monoclonal antibody. **(A)** The immunofluorescence assay of MAbs against MEV in infected F81 cells (magnification, 100×). MEV-infected F81 cells were incubated with MAbs 4, 6, 8, 10, and 14 followed by FITC-conjugated secondary antibody. SP2/0 supernatant as a negative control. **(B)** The MEV VP2 protein was run for Western blotting using one of the MAbs 4, 6, 8, 10, and 14. SP2/0 supernatant and specific anti-MEV MAb F5 (Wang et al., 2009) served as negative and positive controls, respectively.

Positive hybridoma cells were detected, designated as 4, 6, 8, 10, and 14, by indirect ELISA and cloned by limiting dilution. The specificity of generated MAbs to MEV was determined using an indirect immunofluorescence assay (IFA) and Western blotting. The results of the IFA showed that all five MAbs reacted with the MEV-infected F81 cells (Figure 2A), while no fluorescent signal was visualized in negative control with SP2/0 cells suspension. Western blotting results showed that these five MAbs specifically recognized the MEV VP2 protein bands *via* SDS-PAGE, while we used the identified MEV specific monoclonal antibody MAb F5 (Wang et al., 2009) as the negative control (Figure 2B). The two assays demonstrated that these antibodies had good specificity and the MAb 14 was selected for preparation of the ICS assay.

### Characterization of the Colloidal Gold Probes

**Figure 3:**
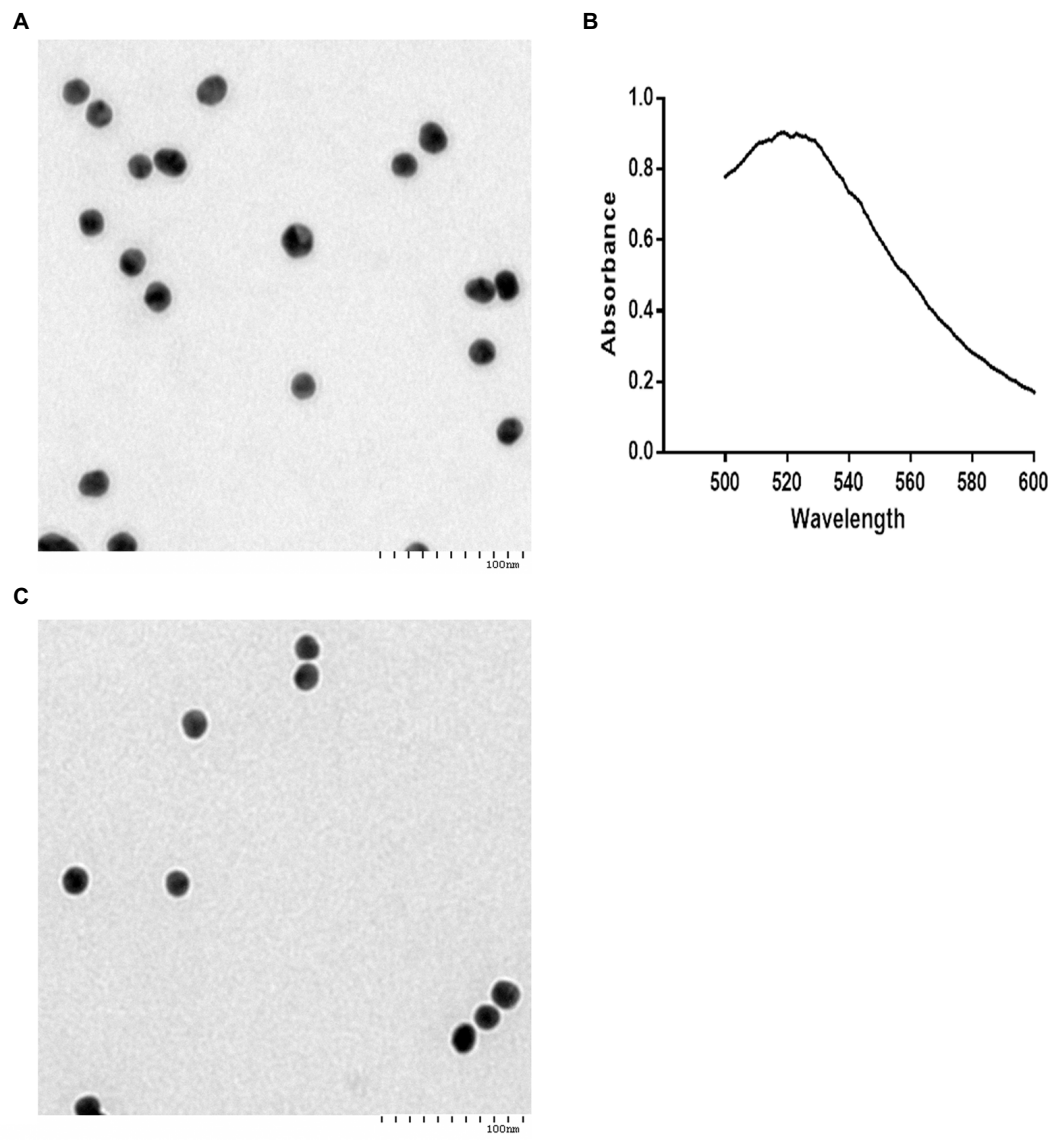
Characterization of the colloidal gold probes. Analysis of the 20-nm colloidal gold solution **(A,B)**. **(A)** About 20-nm colloidal gold particles appear round or oval observed by transmission electron microscopy (TEM). TEM indicated that the diameters of the colloidal gold particles were approximately 20 nm. Bar = 100 nm. **(B)** Determination of homogeneity of 20-nm colloidal gold solution by UV-visible spectrophotometry. **(C)** Gold colloid-labeled MAb 14 appeared round or oval, and a white ring around the probe, without any sediment, as observed by TEM. Bar = 100 nm.

The 20-nm colloidal gold solution was wine red with good transparency and without sediments or suspended solids (data not shown). When viewed under a TEM, the colloidal gold particles were uniform in size and had good dispersibility (Figure 3A). By UV-visible spectrophotometry, the colloidal gold solution showed a maximum absorption value of approximately 0.9 at 520 nm (Figure 3B). The colloidal gold with the MAb probe was dark red, exhibiting good transparency and dispersibility (data not shown). There was a white ring around the probe without any sediment by TEM (Figure 3C).

### Assay Principle

The test is ICS based on the use of one MEV-MAb 14 antibody, purified MEV VP2 protein, and goat anti-mouse IgG. The test device includes a test strip with a plastic test cassette. The antibodies and protein are attached to three different zones on the membrane; a sample pad, a T line, and a C line. MEV MAb conjugated with colloidal gold particles is attached to the sample pad. Purified MEV VP2 protein is permanently immobilized to the T line and the goat anti-mouse IgG is immobilized to the C line (Figure 1A). In the testing process, 100 μl liquid samples are dropped onto the sample pad, and a C line appears when samples contain MEV. When the sample liquid reaches the conjugate pad, the MEV reacts with the colloidal gold-MAb 14 conjugates to form an antigen-colloidal gold-MAb 14 complex then travels through the nitrocellulose membrane *via* capillary action. The complex could not bind to the MEV VP2 protein on the T line but reacted with goat anti-mouse IgG on the C line, resulting in a dark red band (Figure 1B). Conversely, samples without MEV, the colloidal gold-MAb 14 conjugates could react with both VP2 protein and goat anti-mouse IgG, resulting in two dark red bands on T and C lines (Figure 1C). The tests are invalid if only one red band is present in the T line and no red band is present in the T and C lines (Figure 1D).

### Specificity, Sensitivity, and Stability of the ICS

**Figure 4:**
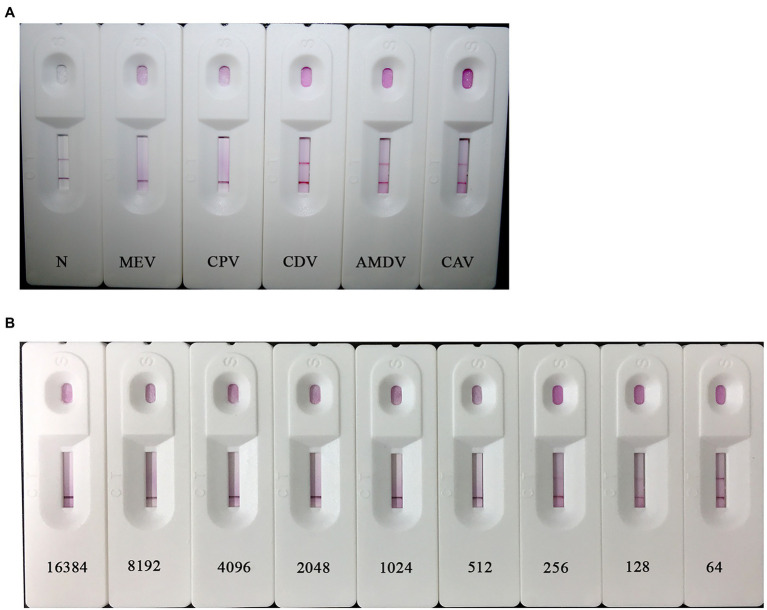
Specificity and sensitivity of the colloidal gold ICS. **(A)** Specificity of the colloidal gold ICS. The MEV, CPV, canine distemper virus (CDV), Aleutian mink disease virus (AMDV), and canine adenovirus (CAV) strains were tested by ICS. All specimens other than MEV and CPV resulted in two bands on the T line and C line. **(B)** Sensitivity test of the ICS. Different concentrations of MEV B strain (MEVB) subjected to MEV ICS (2^14^–2^7^ hemagglutination unit/100 μl, respectively). Similar results were obtained in three independent experiments by different operators (data not shown).

The MEVB and CPV-BJ-21 strains were successfully detected by the MEV ICS, but they did not react with the CDV3, CAV-2c, and AMDV strains, suggesting that the MEV ICS has good specificity (Figure 4A). Two-fold dilutions of the supernatant of the MEVB strain were used for the determination of the sensitivity of the ICS and indicated that the detection limit of ICS was 512 hemagglutination unit of MEVB/μl (Figure 4B). Furthermore, the strips showed no obvious changes in the sensitivity and specificity after storage at 4 or 25°C for 6 months, indicating that they have good reproducibility and stability (data not shown).

### Clinical Application of the ICS

Of the 289 samples, 220 were positive by the rapid CPV Ag test kit, while 207 fecal samples were found to be positive by the MEV ICS. Of these, 205 fecal samples (70.9%) were positive by the rapid CPV Ag test kit and the MEV ICS, and 67 samples (23.2%) were negative by both the rapid CPV Ag test kit and the MEV ICS (Table 1). Compared with the commercial kit, the percentage of agreement, relative sensitivity, and relative specificity of the MEV ICS were 94.1% (272/289), 93.2% (205/220), and 97.1% (67/69), respectively.

**Table 1:** Comparison of the sensitivity and specificity of the mink enteritis virus (MEV) immunochromatographic strip (ICS) and canine parvovirus (CPV) commercial kit (ICS, BioNote, Korea) for detection of MEV/CPV in fecal samples.

MEV ICS	CPV ICS		Total
	Positive	Negative	
Positive	205	2	207
Negative	15	67	82
Total	220	69	289

Percentage of agreement: (205 + 67)/289 = 94.1%; relative sensitivity: 205/(205 + 15) = 93.2%; and relative specificity: 67/(67 + 2) = 97.1%.

## Discussion

Mink enteritis virus is responsible for causing viral enteritis in the fur industry worldwide and is known for its high pathogenicity (Steinel et al., 2001). As it spreads rapidly, timely diagnosis of MEV is important for the prevention and control of MEV infection (Sun et al., 2013). Currently, there is no on-site detection for MEV, and its diagnosis requires laboratory equipment (Wang et al., 2013). Although Esfandiari and Klingeborn (2000) have developed a one-step immunochromatographic test based on monoclonal antibody and latex particles for the diagnosis of canine parvovirus. They used the CPV-MAbs and latex particles, which means the method has false-negative results for some MEV isolates that have a low identity to CPV. To the best of our knowledge, this is the first report of on-site detection for MEV by colloidal gold test strip, with only 100 μl of liquid sample for each test and it can be detected in approximately 2 min, greatly reducing the time required for diagnosis. This strip can be used outside the laboratory setting and is relatively inexpensive.

Here, the researchers used the competition method to create a colloidal gold strip for the rapid diagnosis of MEV. It can react with both MEV and CPV and does not react with other pathogens that are commonly encountered in mink, such as CDV, ADV, and AMDV. This means that the ICS can be used as a tool to detect CPV and MEV in clinical settings. Although both the prepared colloidal gold strip and the commercially available Ag test strip can be used to detect MEV, the colloidal gold test strip should be used due to species-specific differences between CPV and MEV. Compared with the commercially available strip, the percentage of agreement of the MEV ICS is high. Currently, this new method provides an effective supplementary method to carry out epidemiological surveys and eliminate MEV.

Mink enteritis virus and CPV belong to a single species, *Carnivore protoparvovirus 1*, and share common antigenic features. The capsid of the two viruses contains 60 protein subunits of VP1 (5–6 copies) and VP2 (54–55 copies), and those share a common structure. There are two antibody recognition sites, named “A” and “B,” on the capsid (Callaway et al., 2018; Decaro et al., 2020). We think the MEV MAb 14 used in the study can recognize the same site (“A” or “B”) of the MEV and CPV, therefore, the ICS can be used for both MEV and CPV detection. Previously two studies have shown that the CPV-specific MAbs can recognize and react with both CPV and MEV based on the serological assays (Parrish, 1990).

The MEV ICS was developed using specific MAb against the VP2 protein of MEV to identify MEV, which causes significant losses in the fur animal breeding industry worldwide. This method is useful for the early detection of MEV infection in minks by on-site analysis or in combination with other assays. Therefore, this new technique is a valuable advancement in the epidemiological investigation of MEV.

## Conclusion

This study provided an on-site ICS for the rapid detection of MEV. It is very suitable for the detection of MEV in the field, and it can also be used for the diagnosis of CPV in dog populations.

## Data Availability Statement

The original contributions presented in the study are included in the article/supplementary material, further inquiries can be directed to the corresponding authors.

## Ethics Statement

The animal study was reviewed and approved by Ethical Committee of the Institute of Special Animal and Plant Sciences, Chinese Academy of Agricultural Sciences.

## Author Contributions

SC and ZW designed the study. PL and JW performed the experimental work with the help of SS, YC, and LY, collected the sample, and analyzed the data. All authors read and approved the final manuscript.

## Funding

The study was supported by the Central Public-Interest Scientific Institution Basal Research Fund (1610342020044), the Talent Introduction Research Fund of Hebei Agricultural University (YJ2021019), the Capital Construction Funds Planned Projects in the Provincial Budget of 2020 (2020C035-1) and Jilin Province Scientific and Technological Program (20190301086NY and 20200402036NC).

## Conflict of Interest

The authors declare that the research was conducted in the absence of any commercial or financial relationships that could be construed as a potential conflict of interest.

## Publisher’s Note

All claims expressed in this article are solely those of the authors and do not necessarily represent those of their affiliated organizations, or those of the publisher, the editors and the reviewers. Any product that may be evaluated in this article, or claim that may be made by its manufacturer, is not guaranteed or endorsed by the publisher.
